# Hemodynamic Study of a Patient-Specific Intracranial Aneurysm: Comparative Assessment of Tomographic PIV, Stereoscopic PIV, *In Vivo* MRI and Computational Fluid Dynamics

**DOI:** 10.1007/s13239-021-00583-2

**Published:** 2021-11-08

**Authors:** Xiaolin Wu, Stefanie Gürzing, Christiaan Schinkel, Merel Toussaint, Romana Perinajová, Pim van Ooij, Saša Kenjereš

**Affiliations:** 1 Department of Chemical Engineering, Faculty of Applied Sciences, Delft University of Technology, Delft, The Netherlands; 2 J. M. Burgerscentrum Research School for Fluid Mechanics, Delft, The Netherlands; 3 Department of Radiology and Nuclear Medicine, Amsterdam UMC, University of Amsterdam, Amsterdam, The Netherlands

**Keywords:** Intracranial aneurysm, Hemodynamics, Wall shear stress, Particle image velocimetry, Computational fluid dynamics, 4D Flow MRI

## Abstract

**Introduction:**

Wall shear stress (WSS) is associated with the growth and rupture of an intracranial aneurysm. To reveal their underlying connections, many image-based computational fluid dynamics (CFD) studies have been conducted. However, the methodological validations using both *in vivo* medical imaging and *in vitro* optical flow measurements were rarely accompanied in such studies.

**Methods:**

In the present study, we performed a comparative assessment on the hemodynamics of a patient-specific intracranial saccular aneurysm using *in vivo* 4D Flow MRI, *in silico* CFD, *in vitro* stereoscopic and tomographic particle imaging velocimetry (Stereo-PIV and Tomo-PIV) techniques. PIV experiments and CFD were conducted under steady state corresponding to the peak systole of 4D Flow MRI.

**Results:**

The results showed that all modalities provided similar flow features and overall surface distribution of WSS. However, a large variation in the absolute WSS values was found. 4D Flow MRI estimated a 2- to 4-fold lower peak WSS (3.99 Pa) and a 1.6- to 2-fold lower mean WSS (0.94 Pa) than Tomo-PIV, Stereo-PIV, and CFD. Bland-Altman plots of WSS showed that the differences between PIV-/CFD-based WSS and 4D Flow MRI-based WSS increase with higher WSS magnitude. Such proportional trend was absent in the Bland-Altman comparison of velocity where the resolutions of PIV and CFD datasets were matched to 4D Flow MRI. We also found that because of superior resolution in the out-of-plane direction, WSS estimation by Tomo-PIV was higher than Stereo-PIV.

**Conclusions:**

Our results indicated that the differences in spatial resolution could be the main contributor to the discrepancies between each modality. The findings of this study suggest that with current techniques, care should be taken when using absolute WSS values to perform a quantitative risk analysis of aneurysm rupture.

**Supplementary Information:**

The online version contains supplementary material available at 10.1007/s13239-021-00583-2.

## Introduction

Intracranial aneurysm is an abnormal localized enlargement of an artery in the cerebral vasculature. It is estimated that between 1 and 5% of the general population are affected by this condition and that 20-30% of the affected population have multiple aneurysms.^[2,37,28]^ The rupture of an intracranial aneurysm can cause subarachnoid haemorrhage (SAH), which has a high mortality rate of almost 50%.^[1]^ On the other hand, the clinical treatment of an unruptured aneurysm also comes with risks.^[32]^ Therefore, once detected, a rupture risk analysis of the existing aneurysm is important.

Hemodynamics can potentially help with the risk analysis of aneurysm rupture.^[7,8]^ To reveal the underlying connection between hemodynamics and the progression of intracranial aneurysms, various parameters have been studied in the literature. Among them, wall shear stress (WSS) has attracted extensive attention. The accurate calculation of WSS requires three-dimensional three-components (3D3C) velocity data with high spatiotemporal resolutions. Current non-invasive medical techniques such as phase-contrast magnetic resonance imaging (PC-MRI) can provide *in vivo* insights into the local blood flow and WSS distribution.^[35,27,34]^ However, the accuracy of WSS *in vivo* estimation can be limited by imaging noise, artefacts, and relatively low spatiotemporal resolutions available—especially in smaller blood vessels commonly encountered in the brain vasculature.^[29]^

Over the past decades, computational fluid dynamics (CFD) has been extensively applied in various arterial flows due to their ability to simulate instantaneous 3D velocity field and corresponding WSS.^[9,3,39,20]^ Especially with the advancement of medical imaging in recent years, image-based CFD can be applied in patient-specific studies, making it a potential tool for assisting clinical decision-making in the future.^[12]^ However, uncertainties due to imposed modelling assumptions and variations of solution strategies in CFD produced controversial reports, such as the aneurysm rupture is caused by either high or low WSS.^[31,19,38,10,23]^ This is one of the reasons that hinder the clinical translational value of hemodynamics. Hence, the validation of CFD results through either *in vitro* or *in vivo* measurements is necessary. Nevertheless, limited computer simulations results, particularly the CFD-based WSS, have been experimentally verified. Optical imaging measurement techniques, such as particle image velocimetry (PIV), are often used for *in vitro* validations because they provide well-controlled, high-resolution flow fields. The challenge is that the three-dimensional WSS requires a complete velocity gradient tensor, which did not become achievable in optical experiments until the last decade followed by the developments of the full 3D measurement techniques, such as multi-plane stereoscopic PIV (Stereo-PIV), tomographic PIV (Tomo-PIV) and Shake-the-Box (STB).^[5,40,13,4]^

In the current active research area of fluid mechanics in biomedical applications, there is a shift of focus from single- to multi-modality studies. Integrating data (namely *in vivo*, *in vitro*, and *in silico* datasets) is one of the recommended approaches for validating numerical results and providing comprehensive hemodynamic assessments.^[28]^ To date, only one multi-modality study has performed a comparison of WSS obtained by *in vivo* 4D Flow MRI, CFD, and *in vitro* STB techniques under pulsatile condition.^[4]^ However, the high cycle-to-cycle flow variations under pulsatile flow can cause large differences in flow measurements between different modalities,^[22]^ which will amplify the variations in velocity-derived parameters. To further bridge this gap and exclude the cycle-to-cycle hemodynamic variations, we investigated hemodynamics in a patient-specific intracranial aneurysm under steady flow conditions using a multi-modality approach. We compared the steady flow results of PIV (Tomo-PIV, Stereo-PIV) and CFD to the peak systole measurement of *in vivo* 4D Flow MRI. The geometry for PIV and CFD models was based on the 4D Flow MRI velocity field. Steady-state PIV and CFD studies were performed with inlet flow based on *in vivo* 4D Flow MRI at peak systole. In this study, we presented the similarities and differences in velocity field, vortex, and WSS distributions obtained by 4D Flow MRI, Stereo-PIV, Tomo-PIV, and CFD.

## Methods

### PC-MRI Setup

**Figure 1:**
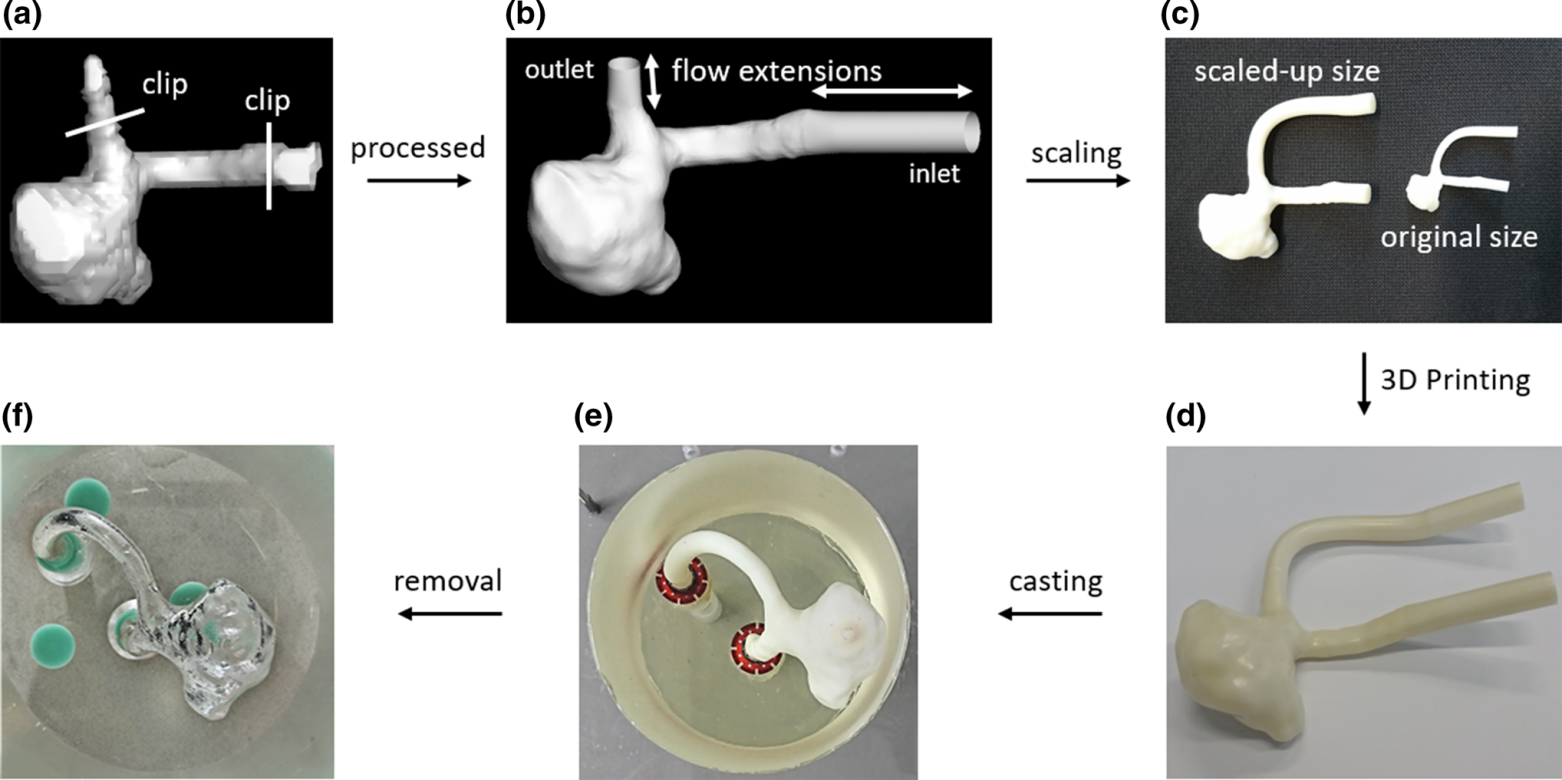
(a) Segemented raw surface from *in vivo* 4D Flow MRI. (b) Processed surface for *in silico* CFD model. (c) Scaling up the geometry for *in vitro* PIV model (Note: for the convenience of casting, the outlet was further extended for *in vitro* PIV model). Manufacturing steps of the PIV phantom: (d) the 3D rapid prototyping printed mould, (e) the mould casted with PDMS, (f) the PDMS phantom with mould removed.

The patient-specific intracranial aneurysm (Fig. 1a) is located at the right middle cerebral artery (RMCA) of the Circle of Willis (CoW). The geometry was reconstructed from the 4D Flow 7T MRI scans performed at the Academic Medical Center in Amsterdam. The patient (man, 65 years old) underwent a 4D Flow MRI examination on a 7T MRI scanner (Achieva, Philips Healthcare, Cleveland, USA) that was retrospectively gated with a peripheral pulse unit. The overall scan time of the PC-MRI measurement was approximately 15 minutes. The sequence is the same as in Ref. [36]. The acquired spatial resolution was 0.47 × 0.47 × 0.5 mm^3^ and the echo time, repetition time and the flip angle were 3.1 ms, 6.8 ms and 20°, respectively. Velocity encoding was 150 cm/s in the x, y, and z directions. The number of reconstructed cardiac phases was 9, resulting in a temporal resolution of 82 ms at an average heart rate of 81 beats/minute. The scan was accelerated with a SENSE factor of 3 in the right-left direction. Phase images were corrected for concomitant field and eddy current related phase offsets. The lumen of the intracranial aneurysm was semi-automatically segmented using commercial software (Mimics, Materialise, Leuven, Belgium).^[35]^ From now on, we will refer to the *in vivo* 4D Flow MRI data as MRI for short.

### Preparations for *In Vitro* and *In Silico* Models

Before being applied to PIV and CFD studies, the segmented raw surface was processed using Vascular Modelling Toolkit (VMTK). Firstly, we applied Taubin smoothing with passbands of 0.45 and 100 iterations. Afterward, the geometry was clipped at the inlet and outlet of the aneurysm to open the inlet/outlet. Flow extensions and caps were subsequently added to the inlet and the outlet. At this stage, the resulting surface (STL) (Fig. 1b) was ready for numerical meshing. For PIV experiments, the STL surface was scaled up 3.77 times to increase the spatial resolution (Fig. 1c). Moreover, the outlet vessel was further extended for the convenience of PIV phantom manufacturing (Fig. 1c). The PIV phantom (Fig. 1f) was constructed by the lost core casting technique. More specifically, a transparent aneurysm phantom was made by casting a removable mould of the aneurysm lumen with PDMS (Polydimethylsiloxane) liquid (Fig. 1e). The mould was a 3D ABS (Acrylonitrile Butadiene Styrene) print fabricated by Fortus 450MC (Stratasys, Israel) machine with a layer resolution of 0.127 mm (Fig. 1d). Once the PDMS had cured, the mould was dissolved by acetone.

### PIV Setup

#### Flow Loop

The experiment was conducted under the steady flow condition, which corresponded to the averaged peak systole of 9 cardiac cycles measured by 4D Flow MRI *in vivo*. To ensure dynamic similarity, the characteristic inlet Reynolds number (*Re* = 350) was imposed. The working fluid in PIV studies is a mixture of water-glycerol with a measured density (DMA 4100 M, Anton Paar, Austria) of *ρ* = 1147 kg/m^3^ and dynamic viscosity of *µ* = 0.008113 Pa s at 25 °C. The water-glycerol mixture recipe was chosen to reduce the optical distortion at the interface of working fluid and PDMS phantom. By changing the water/glycerol concentration, the refractive index (*n* = 1.4107, Bleeker Zeist Holland refractometer) was matched with that of the PDMS phantom to achieve no visible refraction.

**Figure 2:**
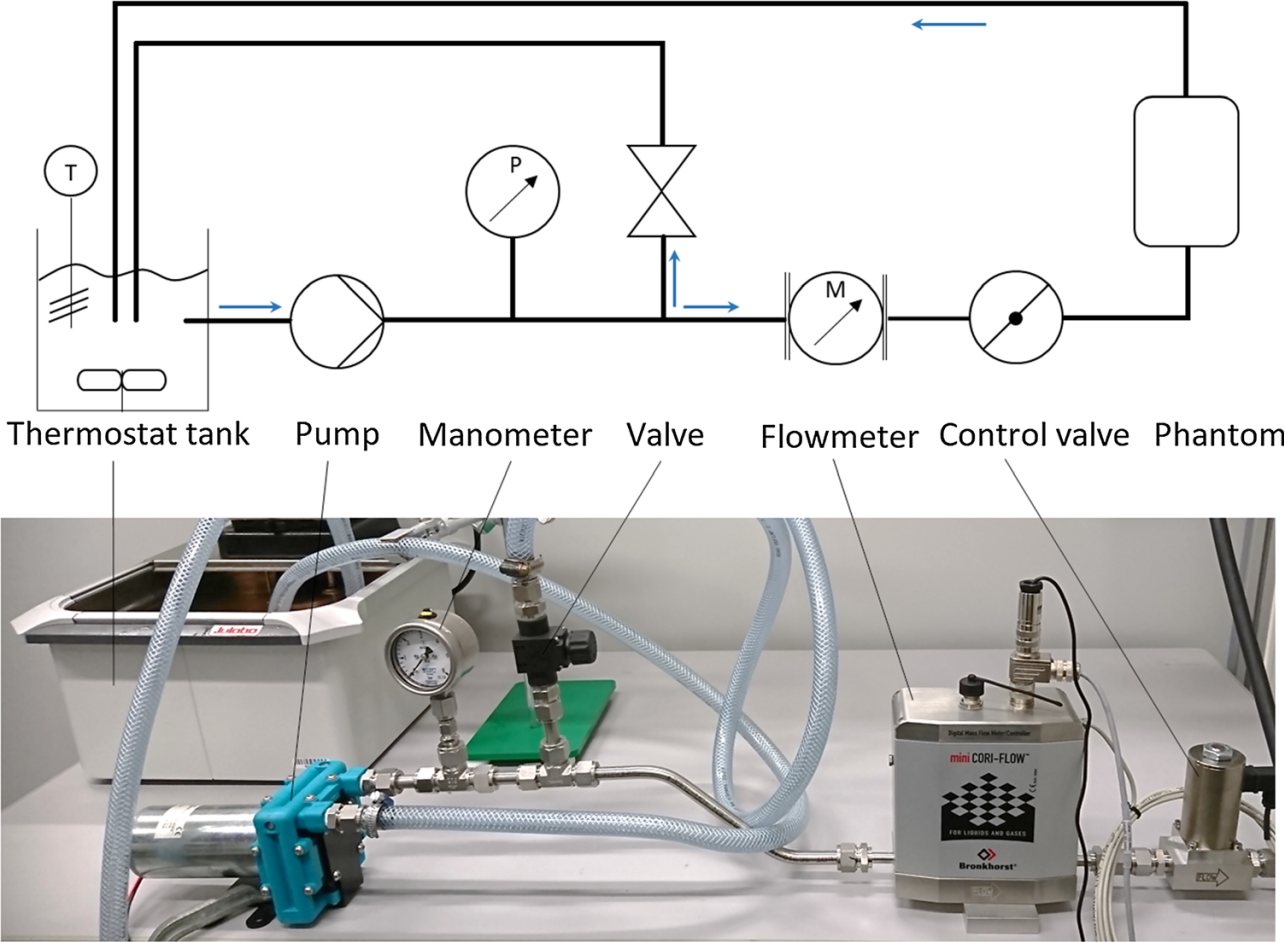
Schematic representation and a photo of constructed flow circulation system.

The flow circulation in the aneurysm was driven by a diaphragm fluid pump (NF 1.600 KPDC, KNF, Germany) (Fig. 2). A thermostat tank was used to keep the temperature of the working fluid at 25 °C. The flow rate was controlled and monitored by an electromagnetic flowmeter (Mini Cori-Flow™ MT5, Bronkhorst, the Netherlands) and a control valve (F-004AC/AI (NC), Bronkhorst, the Netherlands). The inlet flow of PIV experiments measured by the flowmeter was compared to the averaged peak flow measured with 4D Flow MRI. The resulted Reynolds numbers in Stereo-PIV and Tomo-PIV measurements were 327 and 335, which deviated 6.6% and 4.3% from 4D Flow MRI (*Re* = 350), respectively.

#### Imaging System

**Figure 3:**
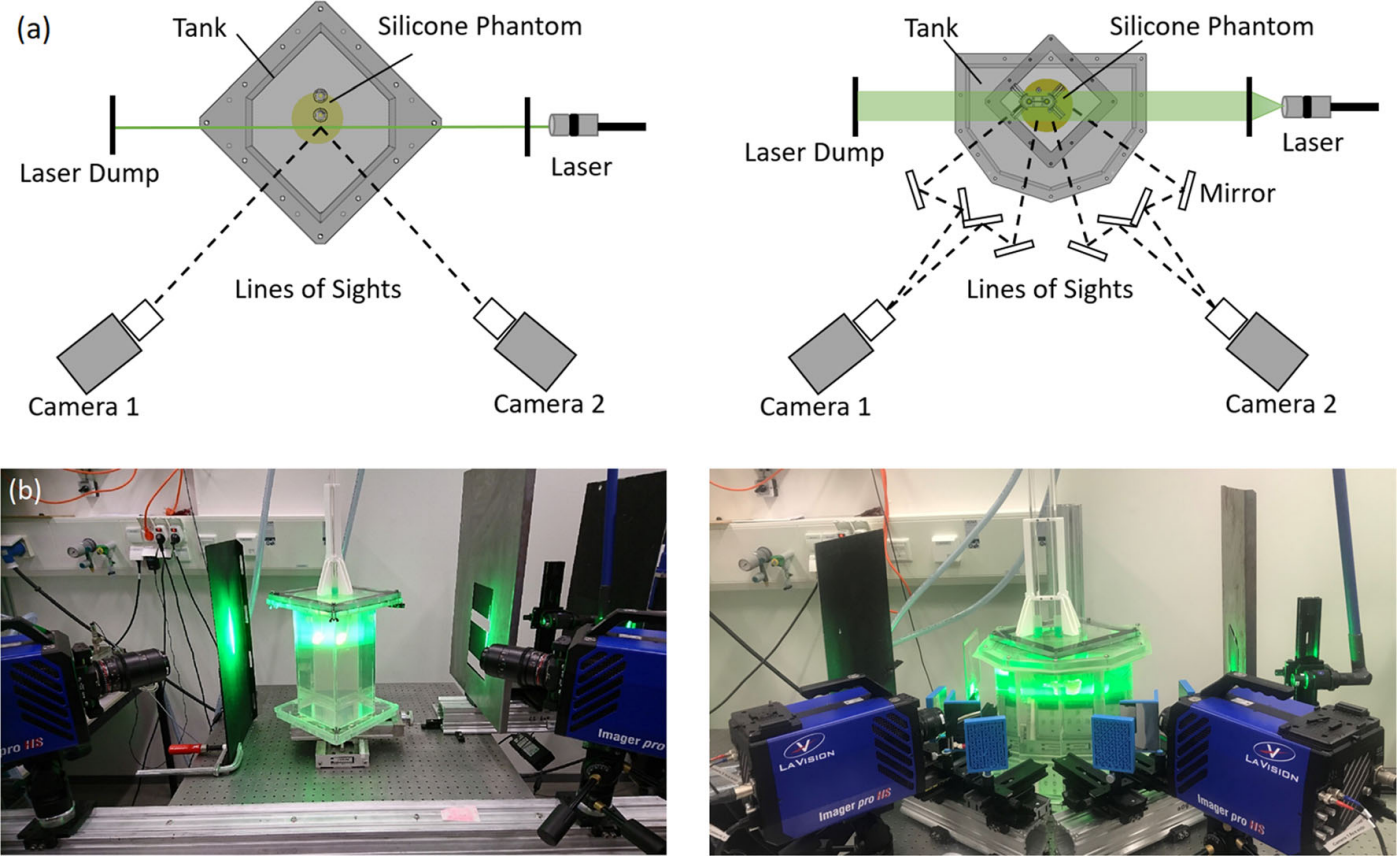
(a) Schematic sketch (top view) of the optical arrangement showing phantom, camera, optics and laser positions. (b) Pictures (front view) of PIV imaging system: left for Stereo-PIV, and right side for Tomo-PIV.

The optical setup of Stereo-PIV and Tomo-PIV used two high-speed CMOS cameras (2016 x 2016 pixel, 12 bit, Imager pro-HS 4M, LaVision Inc, England). In Stereo-PIV, fluorescent dyed polystyrene particles with a diameter of 25 µm and density of 1100 kg/m^3^ was seeded in the flow. In Tomo-PIV, the flow field was seeded with a diameter of 25–50 µm and density of 1100 kg/m^3^ fluorescent Rhodamine-B coated PMMA particles. A volume of approximately 48×70×50 mm^3^ was illuminated by a double pulsed Nd:YLF laser (lDY304, Litron Lasers, England) with the applied laser energy of 27 mJ. A wavelength cut-off filter was equipped on the camera lens to block the laser light. The aneurysm phantom was placed in Plexiglas tanks containing a water-glycerol mixture with the same refractive index of the phantom (Fig. 3). This design of the multi-window tank was to ensure that camera views were orthogonal to the liquid-air interface, reducing optical distortions. For the Stereo-PIV setup, the cameras were arranged in an angular configuration of 90° (Fig. 3). For the Tomo-PIV setup, a mirror system was introduced to create four different views with two cameras. Each camera sensor was split into the left and right half to record two different views. In total, four views were linearly arranged in a horizontal plane with an aperture angle of 108° (Fig. 3). The Plexiglas tanks were mounted on a micrometer slider with an accuracy of ± 0.02 mm (LES4, Isel Germany AG, Eichenzell, Germany), which facilitates the accurate translation of calibrated and measured planes in the z-direction (perpendicular to the laser sheet). Stereo-PIV measurements were taken at 50 parallel planes with a shift of 1 mm to cover the whole aneurysm. In the Tomo-PIV experiment, the entire aneurysm flow field was illuminated and measured at once.

#### Calibration and PIV Analysis

Camera calibrations were performed with a two-level calibration plate placed inside the tank filled with refractive index-matched working fluid. For Tomo-PIV, calibration images at 7 positions equally spaced over 35 mm were taken and used for volumetric calibration along the z-direction. For Stereo-PIV, calibration was done for each measurement plane. A third-order polynomial fitting method was applied to map the 3D world position to the camera sensor plane. This yielded an error of approximately 0.2-0.4 pixel for all views in Tomo-PIV. The volume self-calibration, based on the particle images taken for velocity field calculations, reduced the calibration error to 0.1 pixel, which was necessary for accurate Tomo-PIV reconstruction.^[14]^ Before the volume self-calibration, the image pre-processing of subtracting an average time filter was applied to exclude any tracing particles which stick to the wall. For stereo-PIV, additional self-calibration was also performed to correct misalignment between the calibration plate and the laser sheet.

Before cross-correlation, manual geometric masks were created in all particle images to remove the non-flow regions. Due to the Gaussian laser illumination shape, the masked images were then pre-processed firstly with an intensity normalization filter, subsequently with Gaussian smoothing (3 × 3) and sharpening. In the Stereo-PIV analysis, the cross-correlation started with 2 passes at window size 48 × 48 pixel with 50 % overlap and rectangular weight. Then it was followed by 3 passes decreased window size 12 × 12 pixel with 75% overlap using a circular weight. The resulting final spatial resolution was 0.144 mm^2^. In the Tomo-PIV analysis, the 3D correlation were performed iteratively with 6 steps and with 2 passes in each step. It started with window size 96 × 96 × 96 voxel (75% overlap) and then deformed to a 2 times smaller window size in the following each time step. The final interrogation window size was 16 × 16 × 16 voxel with 75% overlap, resulting in a spatial resolution of 0.25 mm^3^. All vector fields were post-processed by a median filter (5 × 5) for the outlier detection, removal, and replacement. In addition, parts with a correlation value of < 0.8 were removed. The removed parts were filled up by averaging 100 vector fields. The PIV processing was performed using DAVIS 10.0.5. The 2D velocity results of Stereo-PIV were extended to 3D by interpolating the velocity vectors on 50 planes into a rectangular field.

### CFD Simulation

The CFD simulations were carried out with the finite-volume code ANSYS Fluent 17.1 (Ansys Inc., Canonsburg, PA, United States). The STL geometry was meshed using the ICEM CFD (Ansys Inc., Canonsburg, PA, United States). To properly resolve the boundary layer, the mesh in the proximity of the wall was composed of 12 layers of flat polyhedral elements, with a first-layer thickness of 0.05 mm and an exponential growth rate of 1.2. In the central part of the domain, polyhedral elements were applied. In total, the mesh contained 0.8 million polyhedral cells. The mean WSS difference between this and a finer mesh of 2.25 million cells simulations was less than 1% confirming that our results are sufficient to obtain grid-independent solutions. Blood was modelled as a Newtonian fluid with a density of 1060 kg/m^3^ and a dynamic viscosity of 0.0035 Pa s. A rigid wall with no-slip conditions was assumed at the aneurysm wall. The steady flow simulation was performed by solving the incompressible 3D Navier-Stokes equations with the solver settings in Table 1. A parabolic velocity profile was imposed at the inlet and the outlet was set to the outflow boundary condition, with a zero diffusion flux for all flow variables.

**Table 1:** The CFD solver (ANSYS/ Fluent) settings.

Settings	Method/Value
Physics solver	Pressure based
Pressure	Second-order upwind
Momentum	Second-order upwind
Gradient	Least square cell-based
Velocity/pressure coupling	SIMPLE
Convergence criterion	10^-6^

### Calculation of Wall Shear Stress

WSS was estimated by mapping the velocity gradient from the measured/simulated data to the STL surface grids. As mentioned previously, the STL surface was based on MRI velocity field with a resolution of 0.47 × 0.47 × 0.5 mm^3^. The unit inward normal vectors at each surface point were calculated. The velocity gradients obtained by each modality were mapped from the measured fields to the aneurysm surface by inversed-distance weighted interpolation. The value at each surface point was weighted by a function of the distance between each measured data point to the surface data point. The WSS vectors were computed as:

$$\vec{\tau } = 2\mu \dot{\varepsilon } \cdot \vec{n}$$

where $\vec{\tau }$ is the WSS, $\vec{n}$ is the surface unit normal vector, and $\dot{\varepsilon }$ is the rate of deformation tensor, which is calculated as

$$\dot{\varepsilon }_{ij} = \frac{1}{2}\left( {\partial_{j} u_{i} + \partial_{i} u_{j} } \right)$$

where indices *i*, *j* = [1–3] are for the 3 Cartesian (x-y-z) coordinates. In the three-dimensional coordinate system, the WSS vectors were calculated as:

$$\vec{\tau } = \left[ {\begin{array}{*{20}c} {\tau_{x} } \\ {\tau_{y} } \\ {\tau_{z} } \\ \end{array} } \right] = \mu \left[ {\begin{array}{*{20}c} {2n_{x} \frac{{\partial u_{x} }}{\partial x} + n_{y} \left( {\frac{{\partial u_{y} }}{\partial x} + \frac{{\partial u_{x} }}{\partial y}} \right) + n_{z} \left( {\frac{{\partial u_{z} }}{\partial x} + \frac{{\partial u_{x} }}{\partial z}} \right)} \\ {n_{x} \left( {\frac{{\partial u_{x} }}{\partial y} + \frac{{\partial u_{y} }}{\partial x}} \right) + 2n_{y} \frac{{\partial u_{y} }}{\partial y} + n_{z} \left( {\frac{{\partial u_{z} }}{\partial y} + \frac{{\partial u_{y} }}{\partial z}} \right)} \\ {n_{x} \left( {\frac{{\partial u_{x} }}{\partial z} + \frac{{\partial u_{z} }}{\partial x}} \right) + n_{y} \left( {\frac{{\partial u_{y} }}{\partial z} + \frac{{\partial u_{z} }}{\partial y}} \right) + 2n_{z} \frac{{\partial u_{z} }}{\partial z}} \\ \end{array} } \right]$$

where $\tau_{x} ,{ }\tau_{y} ,\tau_{z}$ are the WSS components in the x*-*, y*-* and z*-*direction, while $n_{x} ,{ }n_{y} {\text{ and }}n_{z}$ are corresponding unit wall normals. The WSS calculation method was validated against the analytical solution at the inlet where the flow was parabolic. The error was below 20% for PIV experiments. This error should have included the uncertainties propagated by PIV measurements, the uncertainty of wall location and the bias in velocity gradient interpolation.

## Results

### Comparison of Flow Patterns

In PIV experiments, the aneurysm size was scaled up and a matched Reynolds number was imposed at the inlet, which ensured similar flow patterns to MRI flow. Consequently, the inflow velocity varied from that of MRI. In order to exclude the impact of varied inflow velocity across modalities, we normalized the velocity with the maximum velocity of the inlet (V_ref_) during comparison of the flow field.

**Figure 4:**
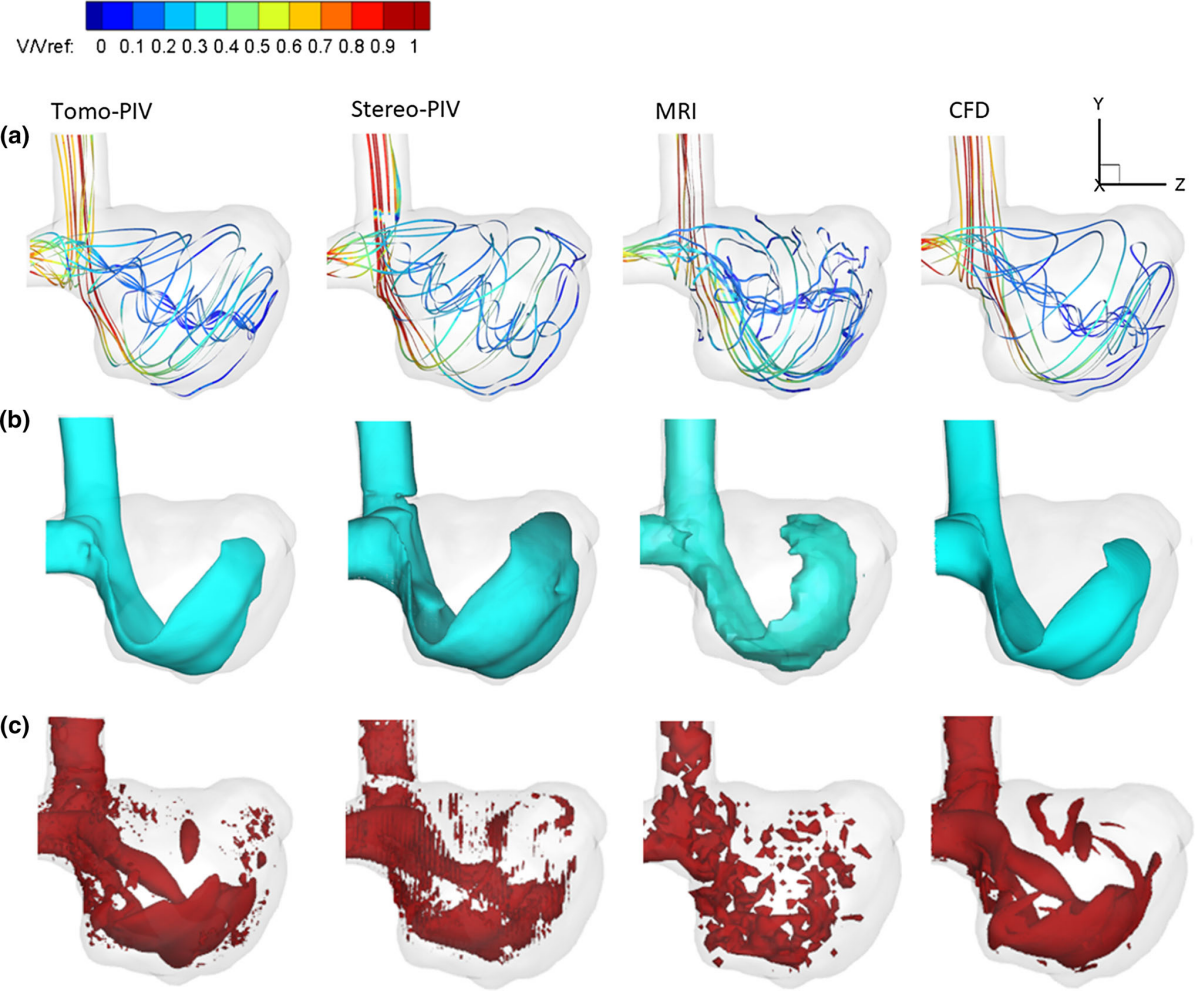
(a) Comparison of streamlines coloured by normalized velocity magnitude acquired by Tomo-PIV, Stereo-PIV, MRI and CFD. (b) Iso-surface of normalized velocity magnitude (V/V_ref_ = 0.3) from all modalities. (c) Comparison of 3D vortex structures identified by *Q*-criterion and visualized with the selected iso-surface (*Q* = 9000 1/s^2^).

The global flow structure obtained by each modality was examined first. The streamlines generated from Tomo-PIV, Stereo-PIV, MRI, and CFD data are shown in Fig. 4a. They are coloured with normalized velocity magnitude. It can be seen that all modalities show the following flow patterns: the inflow forms a wall impingement region and generates bifurcating flow at the neck of the aneurysm; a part of the flow goes directly to the outlet, while the rest of the flow forms a jet that enters the aneurysm sac. We observed discrepancy in the aneurysm core: helical outflow in PIV and CFD results is not detected by MRI.

### Comparison of Velocity Field

The detailed comparisons of the measured and predicted velocity fields were performed next. The iso-surface of normalized velocity magnitude is shown in Fig. 4b. The selected value of normalized velocity iso-surface (V/V_ref_ = 0.3) captures nicely the jet inflow and gives a similar shape for all modalities. Almost identical results were achieved between the Tomo-PIV and CFD. The MRI shows a slightly smaller recirculation angle of the jet inflow. This is commonly found in MRI acquisition due to displacement artefacts.

**Figure 5:**
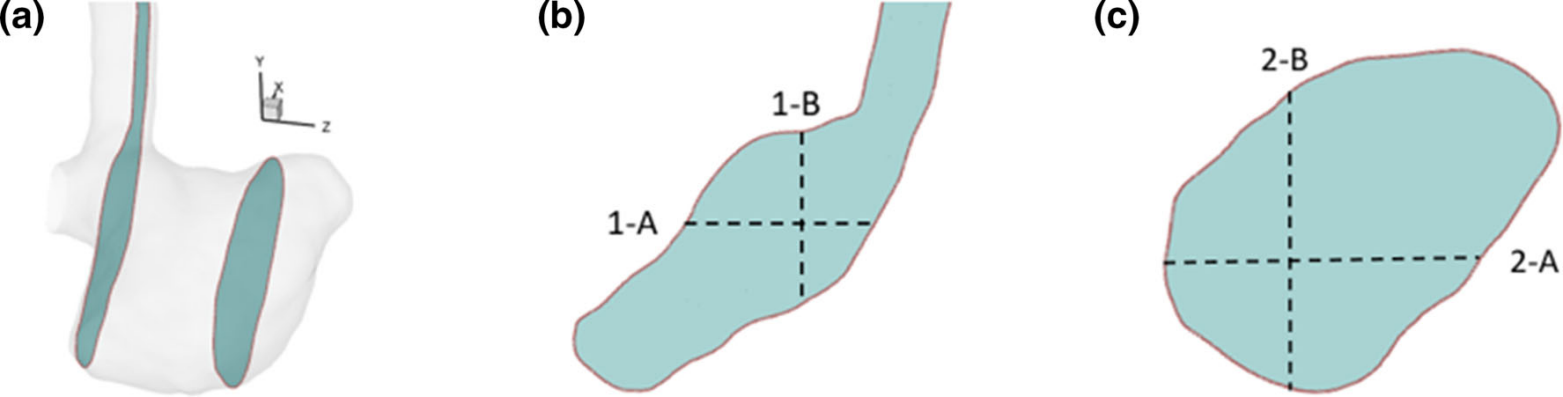
(a) Positions of characteristic 2D cross-sections used for a detailed comparison. (b) and (c) Locations of specific horizontal and vertical lines in the selected cross-sections: (b) 1-A and 1-B, (c) 2-A and 2-B.

**Figure 6:**
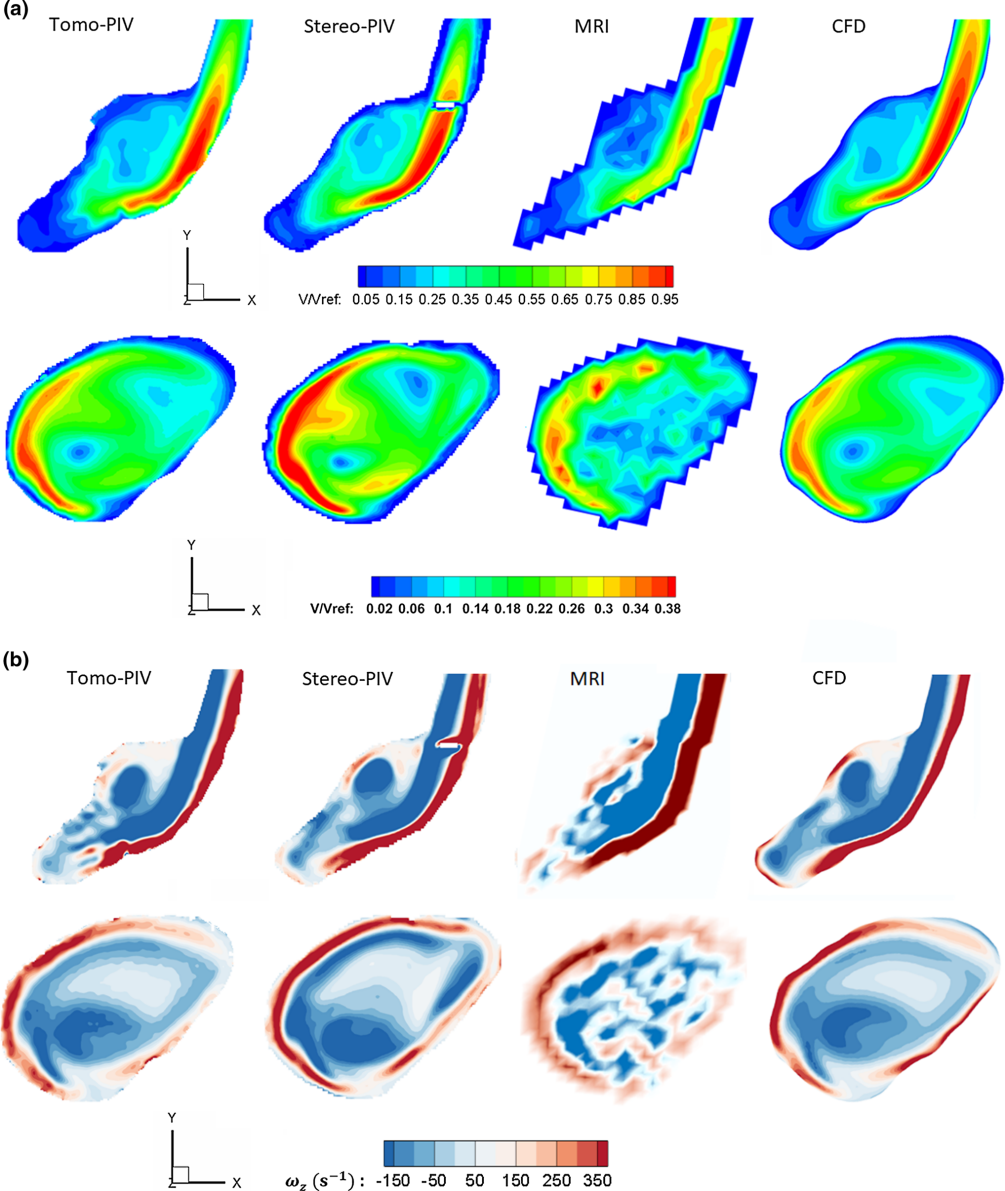
(a) Contours of the normalized velocity magnitude, and (b) contours of the out-of-plane vorticity component in characteristic cross-sections shown in Fig.5a for all modalities. Note: the white area in the stereo-PIV measurements is due to missing data points, the cause was a trapped air bubble

Two characteristic cross-sections along the z-coordinate direction (as indicated in Fig. 5a) were selected to conduct a comparison of velocity magnitude among all modalities. The contours of normalized velocity magnitude in two selected planes are shown in Fig. 6a. It can be seen that a good agreement is obtained between both PIV experiments and CFD. The MRI underestimated the velocity magnitude in the aneurysm core region, where the flow is stagnant and severally distorted. This may be due to the limited resolution of MRI acquisition or measurement errors in those voxels.

**Figure 7:**
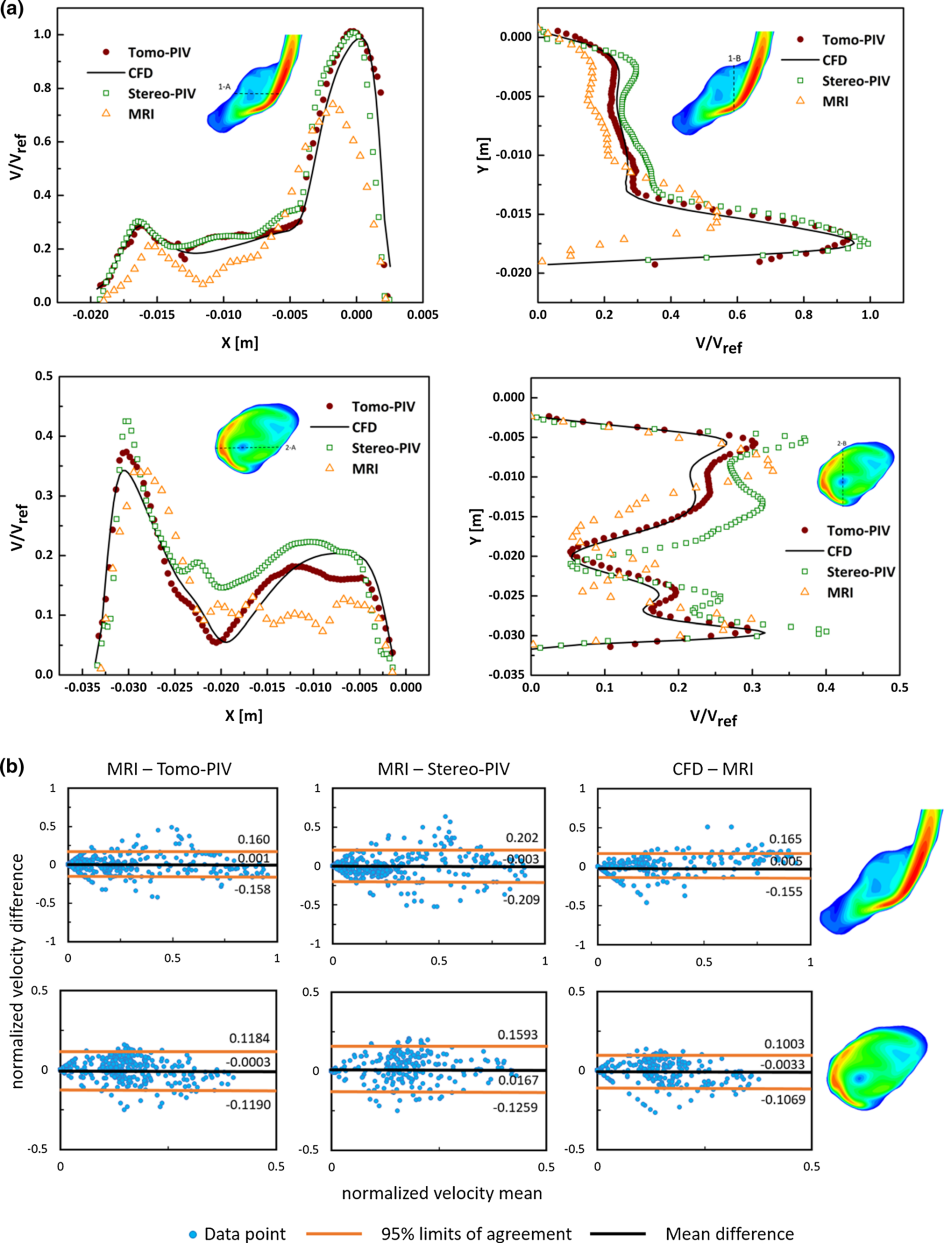
(a) Comparison of Tomo-PIV, Stereo-PIV, MRI and CFD velocity profiles along specific lines in characteristic cross-sections shown in Figs. 5a–5c. (b) Bland-Altman plots of normalized velocity magnitude in two cross-sections, comparing MRI to down-sampled Tomo-PIV, Stereo-PIV and CFD datasets. The 95% limits of agreement were equal to 1.96 SD (where SD is the standard deviation).

To provide a quantitative comparison, profiles of the velocity magnitude along selected horizontal and vertical lines (A and B, as indicated in Figs. 5b and 5c) were extracted in specified cross-sections (Fig. 5a) and shown in Fig. 7a. The corresponding Pearson correlation coefficients are given in Table 2. The velocity profiles exhibit similar trends at both locations for all modalities. Compared to the PIV and CFD, the MRI gives underestimated values of the peak velocity at the 1-A and 1-B locations (see in Fig. 7a–top). The calculated Pearson correlation coefficients (p) of selected profiles show a good agreement between the Tomo-PIV and CFD of 0.95 < p_1D_ < 0.98. The correlation coefficient is lower for the MRI versus CFD comparison, showing a correlation of p_1D_ > 0.67 for most profiles, with the exception of profile 1-B where the MRI significantly underestimated the jet peak value.

**Table 2:** Pearson correlation coefficients (p) of different methods for velocity magnitude profiles along specific lines in characteristic cross-sections shown in Fig. 5.

Location	1-A	1-B	2-A	2-B
Tomo-PIV – CFD	0.98	0.95	0.95	0.96
Stereo-PIV – CFD	0.93	0.98	0.79	0.91
MRI – CFD	0.80	0.44	0.71	0.67

A Bland-Altman analysis was performed to present differences in normalized velocity at the selected planes. To exclude the influence of the spatial resolutions, PIV and CFD datasets were down-sampled to match the MRI resolution by bilinear interpolation. The results are shown in Fig. 7b. It can be seen that the spread of data points was relatively symmetric, and no significant proportional relationship was found between difference and mean values. The mean differences in all Bland-Altman plots are close to 0. The mean differences between MRI and Tomo-PIV are the lowest which is 0.001 and -0.0003 in the first and second plane, respectively.

### Comparison of Vorticity

To further evaluate the ability of different modalities in capturing the 3D flow, the vorticity and *Q*-criterion (the second invariant of the velocity-gradient tensor) as the vortex detection methods were analysed next. To make the out-of-plane vorticity comparable, we scaled the vorticity level by the average velocity and the vessel size at the inlet:

$$\omega_{z} = \left( {\frac{{\partial u_{y} }}{\partial x} - \frac{{\partial u_{x} }}{\partial y}} \right) \times \frac{{u_{{{\text{MRI}}}} }}{{u_{{{\text{PIV}}}} }} \times \frac{{d_{{{\text{PIV}}}} }}{{d_{{{\text{MRI}}}} }}$$

Fig. 4c shows the *Q*-criterion iso-surface and Fig. 6b shows the contours of the out-of-plane vorticity component of all modalities. Again, a good qualitative agreement is obtained between PIV and CFD. The coherent structures developed by the wall jet and helical outflow are clearly captured by PIV and CFD results. Clear discrepancies observed in MRI are: distributions appear less smooth due to lower spatial resolution; the formation of the vortex in the outflow tract was not detected in MRI data.

### Comparison of Wall Shear Stress

As mentioned previously, there was a variation of fluid viscosity, inflow velocity, and vessel size between PIV experiments and MRI/CFD. To compare WSS quantitatively, we scaled the WSS level by:

$$WSS = \tau \times \left( {\mu_{{{\text{MRI}}}} /\mu_{{{\text{PIV}}}} } \right) \times \left( {u_{{{\text{MRI}}}} /u_{{{\text{PIV}}}} } \right) \times \left( {d_{{{\text{PIV}}}} /d_{{{\text{MRI}}}} } \right)$$

**Figure 8:**
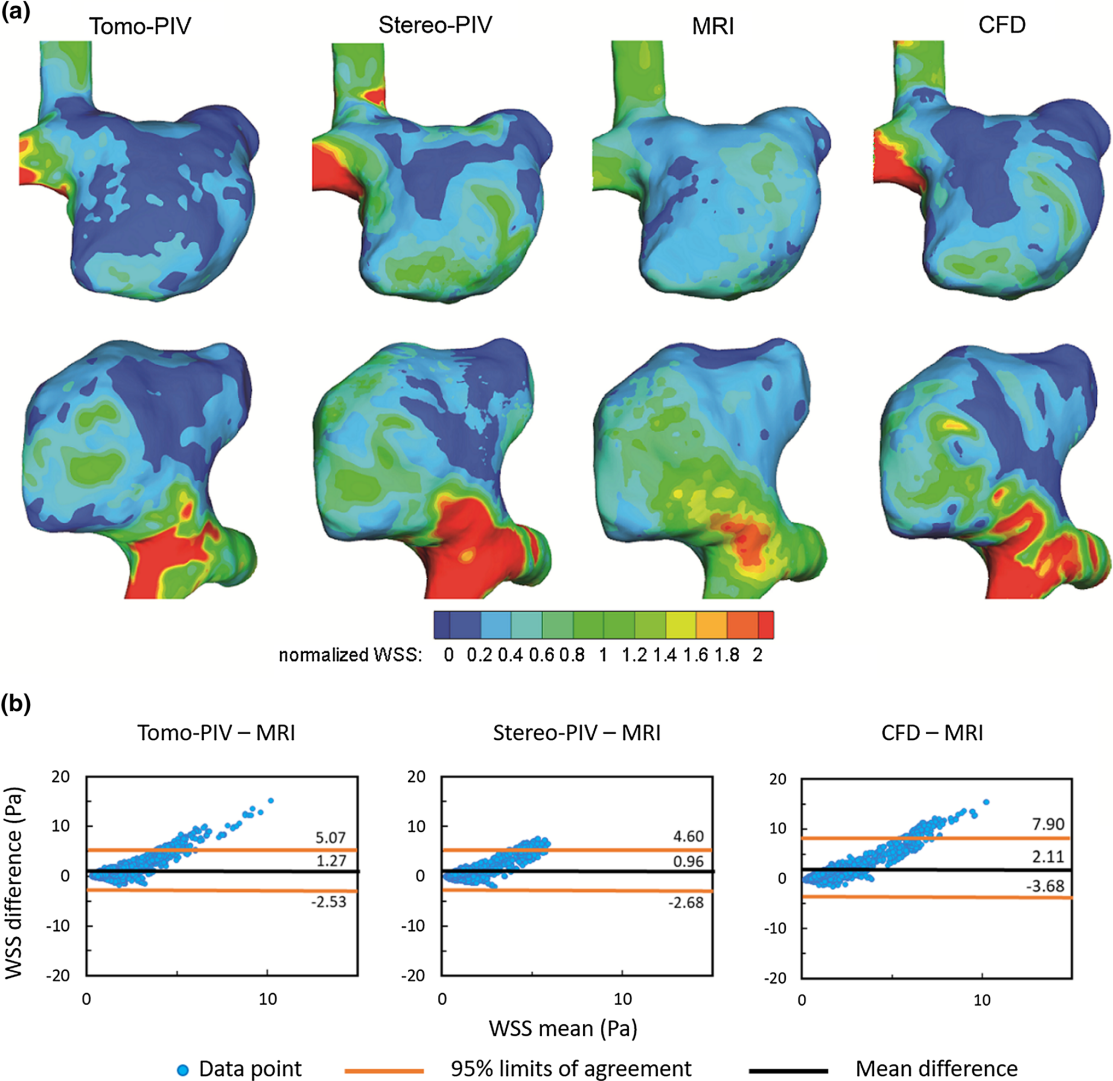
(a) Comparison of normalized WSS distribution between Tomo-PIV, Stereo-PIV, MRI and CFD results. (b) Bland-Altman plots of absolute WSS values, comparing MRI to Tomo-PIV, Stereo-PIV and CFD. The 95% limits of agreement were equal to 1.96 SD (where SD is the standard deviation).

Note that the same method described in section 2.5 and the original spatial resolution of each modality were used for the WSS calculation. For qualitative comparison, the WSS magnitude was normalized by the WSS at the parent vessel of each modality. Figure 8a illustrates the normalized WSS distribution at the aneurysm surface by all modalities. The normalized WSS patterns show a qualitatively reasonable agreement between various modalities. The local distribution of the WSS can be associated closely with the characteristic near-flow structures in the proximity of the wall. The high WSS regions occur along with the inflow jet, whereas the lower WSS regions are present in the rest of the aneurysm wall. In all modalities, the WSS value at the aneurysm neck is more than twice higher than the one in the sac.

Table 3 gives the mean (spatially averaged) and peak absolute WSS magnitude of all modalities. It can be seen that CFD predicts the highest values among all modalities, with peak WSS of 17.91 Pa and averaged WSS of 2.15 Pa. Absolute WSS based on MRI velocity field is over two times smaller than PIV and CFD estimations, which can be attributed to the lower spatial resolution of MRI.^[35]^ The PIV techniques also produce lower values than CFD does, but the differences are less significant. Figure 8b shows the Bland-Altman plots comparing the WSS magnitude derived from Tomo-PIV, Stereo-PIV, CFD to that derived from *in vivo* MRI. The mean difference is 1.27 Pa, 0.96 Pa, 2.11 Pa for Tomo-PIV, Stereo-PIV, and CFD, respectively. The 95% limits of agreement is ± 3.80 Pa, ± 3.64 Pa, and ± 5.79 Pa for Tomo-PIV, Stereo-PIV, and CFD, respectively. For all modalities, the difference increases when the WSS magnitude is higher.

**Table 3:** Mean and maximum WSS in Tomo-PIV, Stereo-PIV, MRI, and CFD.

WSS (Pa)	Tomo-PIV	Stereo-PIV	MRI	CFD
Maximum	17.73	9.32	3.99	17.91
Mean	1.75	1.56	0.94	2.15

## Discussion

The association of WSS and its derivatives with the progression and ultimate rupture of intracranial aneurysms has become a growing interest. In the literature, the WSS studies heavily rely on clinical imaging techniques and CFD simulations. An alternative and increasingly popular technique is Tomo-PIV. Several studies have applied Tomo-PIV in assessing WSS *in vitro* because it can measure the velocity in a volumetric and high-resolution manner.^[5,22]^ Given that each modality has its strong and weak points, combining data obtained from different modalities has been proposed in order to ensure the reliability of the results and to help the realization of hemodynamic analysis in clinical decision-making.^[28]^ In this work, we conducted a multi-modality study based on a patient-specific intracranial aneurysm. The velocity field, vorticity, and WSS magnitude were compared between Tomo-PIV, Stereo-PIV, *in vivo* 4D Flow MRI, and CFD. The results reveal that all modalities can capture the flow characteristic of a high-velocity inflow jet and its accompanying vortex ring structure which recirculates the flow towards the upper part of the aneurysm. MRI shows a slightly smaller recirculation angle compared to other techniques. This is commonly found in MRI acquisition due to displacement artefacts.^[15]^ Regarding the velocity field and vorticity, a good agreement was obtained between CFD and PIV measurements (Fig. 6, Fig. 7). However, MRI displays some clear discrepancies. Compared to CFD and PIV, the velocity magnitude by MRI is smaller in both fast and slow flow areas, and the coherent vortex structure in the core region of the aneurysm (Fig. 4c) is absent. When we down-sampled resolutions of CFD and PIV to that of MRI, the discrepancies have greatly reduced, as can be seen in Fig. 7b. This demonstrates that spatial resolution plays an essential role in causing discrepancies of the velocity magnitude among modalities. This notion is consistent with Ref. 30, which also concluded that PC-MRI leads to systematic underestimation of overall velocity magnitude, and resolution is its major contributor.

The overall normalized WSS distribution shows similar patterns (Fig. 8a) across different modalities. The WSS level elevates at the inflow jet, its impingement, and its recirculating regions. From Fig. 8a, we can see that MRI shows a larger area (green) of elevated WSS at the inflow jet tract, which may imply a more diffused inflow compared to CFD and PIV. Quantitatively, the absolute WSS exhibits significant variations across different modalities (Table 2). The CFD predicts the highest WSS magnitudes (peak 17.91 Pa, mean 2.15 Pa). The MRI estimated WSS magnitude (peak 3.99 Pa, mean 0.94 Pa) is around 2- to 4-fold lower than CFD and Tomo-PIV calculations, and around 2-fold lower than Stereo-PIV calculations. It has been previously reported that WSS magnitude based on MRI measurement is underestimated compared with CFD. The mean WSS of a patient-specific aneurysm was around 2 to 3 times lower for MRI measurement than in CFD simulation as reported in Ref. 35. In Ref. 18, CFD gave 1.63 times higher WSS magnitude than MRI did in 5 patient-specific aneurysm studies. Similar to this study, Natito *et al*. found that MRI greatly underestimated WSS in a study of 15 patients with intracranial aneurysms—5 times lower than the value based on CFD.^[24]^ The degree of MRI-based WSS underestimation is dependent on the actual WSS.^[25]^ The higher WSS is, the more the underestimation is. The proportional trend observed in the Bland-Altman plots (Fig. 8b) confirmed this. The Bland-Altman comparisons in absolute WSS (Fig. 8b) indicate a higher agreement of PIV versus MRI than that of CFD versus MRI. The mean difference and 95% limits of agreement of CFD versus MRI is the largest compared with those of Tomo-PIV/Stereo-PIV versus MRI. Studies have shown that spatial resolution is the most significant factor in the velocity and WSS estimation.^[35,25]^ As Fig. 7b shows, no proportional difference of velocity is found when comparing down-sampled PIV and CFD datasets with MRI. Therefore, we believe that the difference in spatial resolution is the main reason for the discrepancies in the velocity and WSS magnitude between modalities in this study.

In this study, we found that even with a lower in-plane spatial resolution comparing to Stereo-PIV, Tomo-PIV provides a higher WSS estimates and shows better agreement with CFD in velocity field. This could be due to the 4 times higher spatial resolution of Tomo-PIV in the depth direction. For Stereo-PIV, the voxel size in the depth dimension is determined by the thickness of the laser sheet (1 mm in this study). Thus, the averaging effect is severe with low-resolution in the depth direction. It was reported that MRI with a voxel size of 1 mm underestimated WSS by 40% in a noise-free numerical simulation of parabolic flow.^[33]^ We can conclude that the inherently three-dimensionality of Tomo-PIV makes it a preferred technique over Stereo-PIV in hemodynamic investigations, especially when considering pulsatile flow studies. It is very time-consuming to perform volume reconstruction on multiple planes of Stereo-PIV measurement at each time step of the pulsatile cycle.

Several assumptions made in the current PIV and CFD studies, namely the Newtonian viscosity of blood and rigid vessel wall assumptions, could also be associated with the discrepancies. Some numerical studies have reported that neglecting the Non-Newtonian effect of blood and fluid-structure interactions can lead to overestimation of WSS magnitude.^[6,21]^ Only a few comparative experiments studied the impact of fluid-structure interactions on hemodynamics. These experiments were based on rigid and compliant straight vessels and utilized the 2D PIV technique.^[26,17]^ Further experimental data based on patient-specific geometries, non-Newtonian fluid solution, and 3D techniques are required to validate the simulation models. In the current study, the inflow boundary conditions in PIV and CFD are steady, which varies from *in vivo* MRI flow condition. However, we also performed unsteady-state PIV experiments and CFD simulations by imposing a patient-specific waveform at the inlet (see results presented in the Online Resource). The results show that the velocity and WSS distribution at the peak systole are similar to the steady-state. The difference in time-averaged WSS between steady and pulsatile flow condition was studied in Ref. 16 and less than 5% difference was reported. In Ref. 11, steady flow conditions gave lower maximum WSS estimations than pulsatile flow conditions did in a CFD study of 210 cerebral aneurysms. Therefore, we conclude that the imposed steady-state should not be the reason that PIV and CFD predicted higher WSS than MRI measurements. In this study, a rigid transformation was performed in order to compare different datasets. The translational misalignment of coordinate systems can contribute to the qualitative but not quantitative variations of velocity field and WSS among modalities. Further investigation by comparing to more advanced technique such as imaging registration is needed to quantify the impact of geometry mismatch caused by the rigid transformation. In addition, in future work, a combination of compliant model, non-Newtonian fluid, and pulsatile flow condition could bring the *in vitro* and *in silico* studies closer to the real *in vivo* cases.

## Conclusions

We have performed a comparison study of flow and WSS in a patient-specific intracranial aneurysm through *in vivo* 4D Flow MRI, *in vitro* PIV (Stereo-PIV and Tomo-PIV), and *in silico* CFD. Our results demonstrated a good agreement in the flow pattern, velocity, and vorticity between PIV and CFD modalities. However, MRI-based velocity is smaller than velocity based on other techniques in both fast and slow flow areas. The comparison of down-sampled PIV and CFD data to MRI resolution indicates that spatial resolution is the main contributor to the discrepancy. Qualitative agreement in WSS was found across all modalities, but there is a large variation in the absolute WSS values. We observed that the MRI-based WSS magnitude is significantly lower than those based on PIV and CFD. We also found that with a higher out-of-plane spatial resolution, Tomo-PIV gave a higher WSS and better velocity agreement with CFD than Stereo-PIV did. This confirms that spatial resolution plays an important role in the underestimation of WSS. However, the impacts of non-Newtonian viscosity and the compliant wall on WSS should be assessed through *in vitro* experiments in future studies.

## Supplementary Information

Below is the link to the electronic supplementary material.

Supplementary file1 (DOCX 2908 KB)
