# Supplementary material for: Hemodynamic Study of a Patient-Specific Intracranial Aneurysm: Comparative Assessment of Tomographic PIV, Stereoscopic PIV, In Vivo MRI and Computational Fluid Dynamics
Source: Cardiovasc Eng Technol. 2021 Nov 8;13(3):428–42. doi: 10.1007/s13239-021-00583-2 (PMC9197918; doi:10.1007/s13239-021-00583-2)
Supplement: Supplementary file 1 — Supplementary file1 (DOCX 2908 KB) [file 13239_2021_583_MOESM1_ESM.docx]

**Hemodynamic Study of a Patient-specific Intracranial Aneurysm: Comparative Assessment of Tomographic PIV, Stereoscopic PIV, *in vivo* MRI and Computational Fluid Dynamics**

**Xiaolin Wu^1,2^, Stefanie Gürzing^1^, Christiaan Schinkel^1^, Merel Toussaint^1^, Romana Perinajová^1,2^, Pim van Ooij^3^, Saša Kenjereš^1,2, *^**

^1^Department of Chemical Engineering, Faculty of Applied Sciences, Delft University of Technology, Delft, The Netherlands

^2^J. M. Burgerscentrum Research School in Fluid Mechanics, Delft, The Netherlands

^3^Department of Radiology and Nuclear Medicine, Amsterdam UMC, University of Amsterdam, Amsterdam, The Netherlands

***** Correspondence: S.Kenjeres@tudelft.nl

**Supplementary material:** Comparative assessment of the steady and pulsating inflow conditions at the peak systole


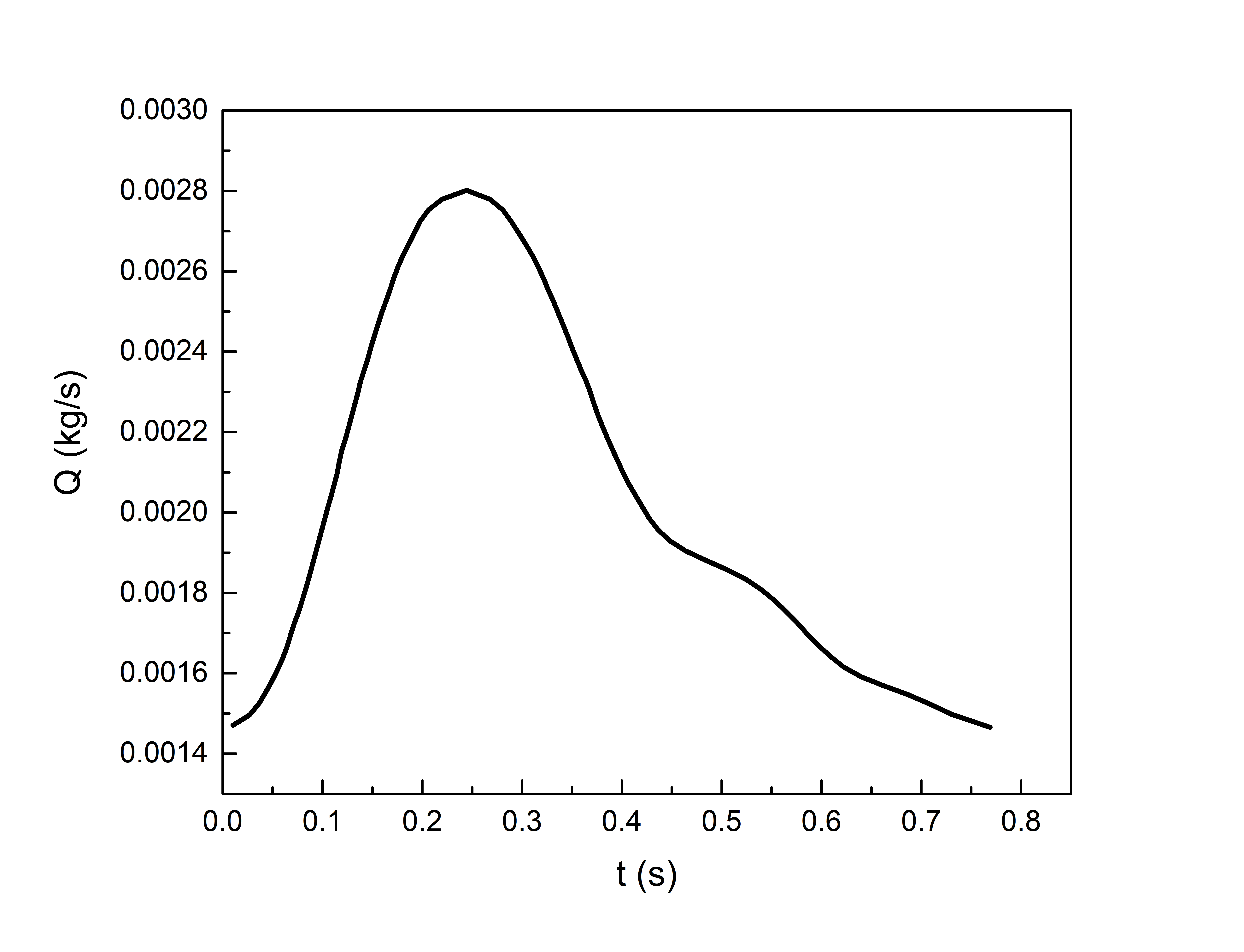

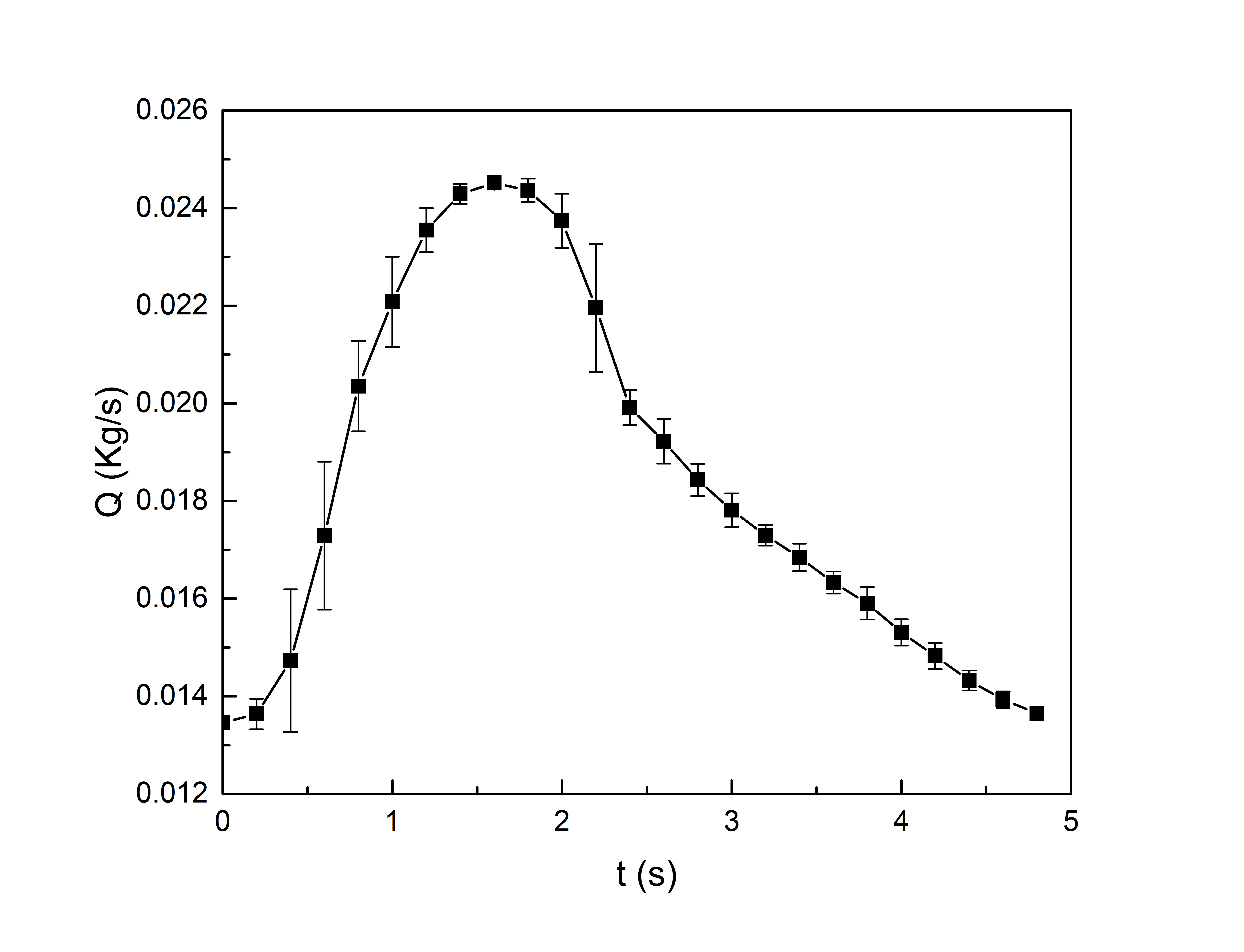


**Fig.1.** Inlet mass flow rates for CFD unsteady simulation (left), and for unsteady PIV measurements (right). The inlet flow profile of CFD was the average flow rate of 9 cardiac cycles measured by 4D Flow MRI. This patient-specific flow pattern was mimicked by the pump system in PIV experiments. Since in PIV experiments the dimension of the aneurysm was scaled up 3.77 times and the fluid properties also differ from blood, the Womersley number (*α* = 2.33) and the Reynolds number (*Re* = 350) which characterize the aneurysmal flow were kept the same as 4D Flow MRI measurement to ensure dynamic similarity.

**
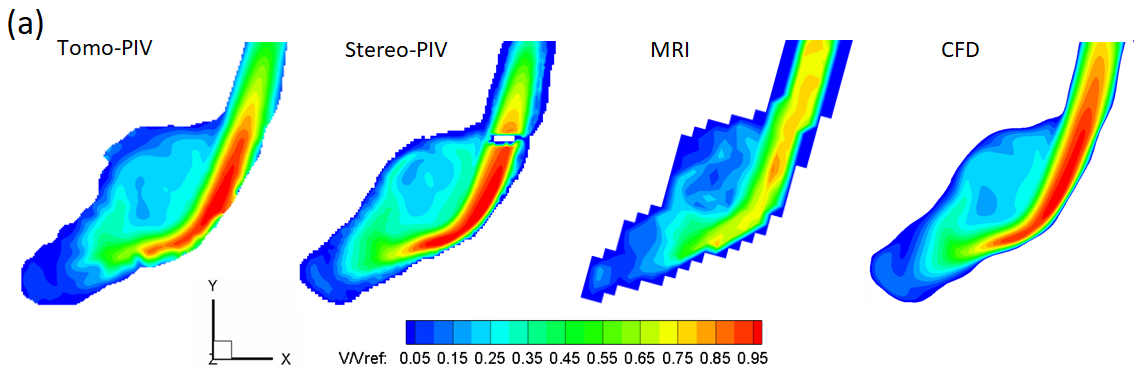
**


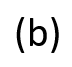
**
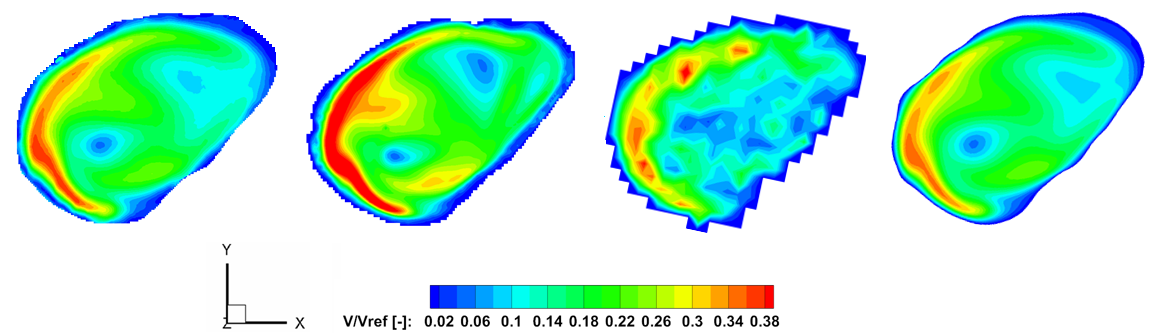
**

**
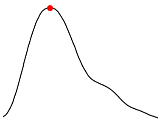
Fig.2.** Velocity magnitude comparison between Tomo-PIV, Stereo-PIV, MRI and CFD at peak systole under (a) steady-state condition and (b) pulsatile flow condition.


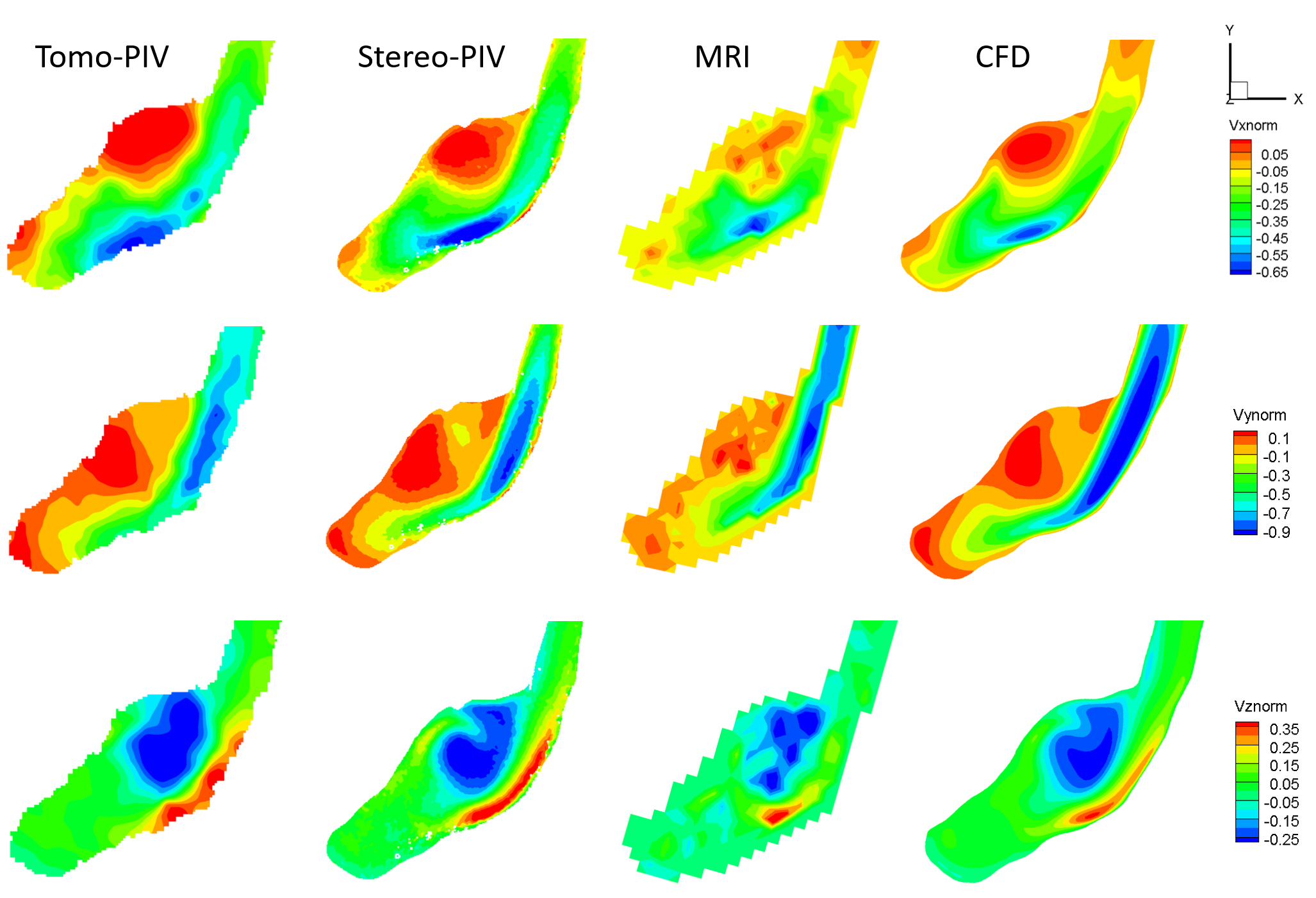


**Fig.3.** Velocity components comparison between Tomo-PIV, Stereo-PIV, MRI and CFD at peak systole of pulsatile flow condition.


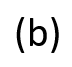

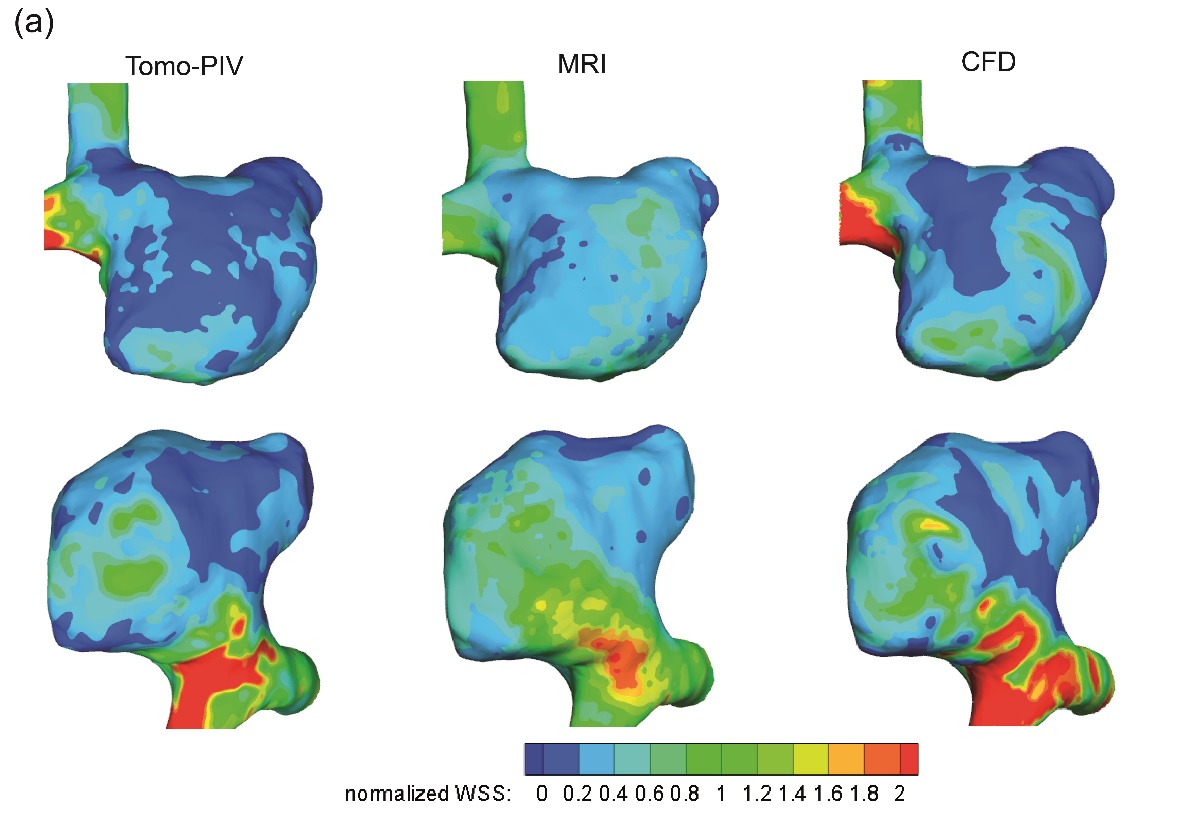


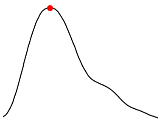

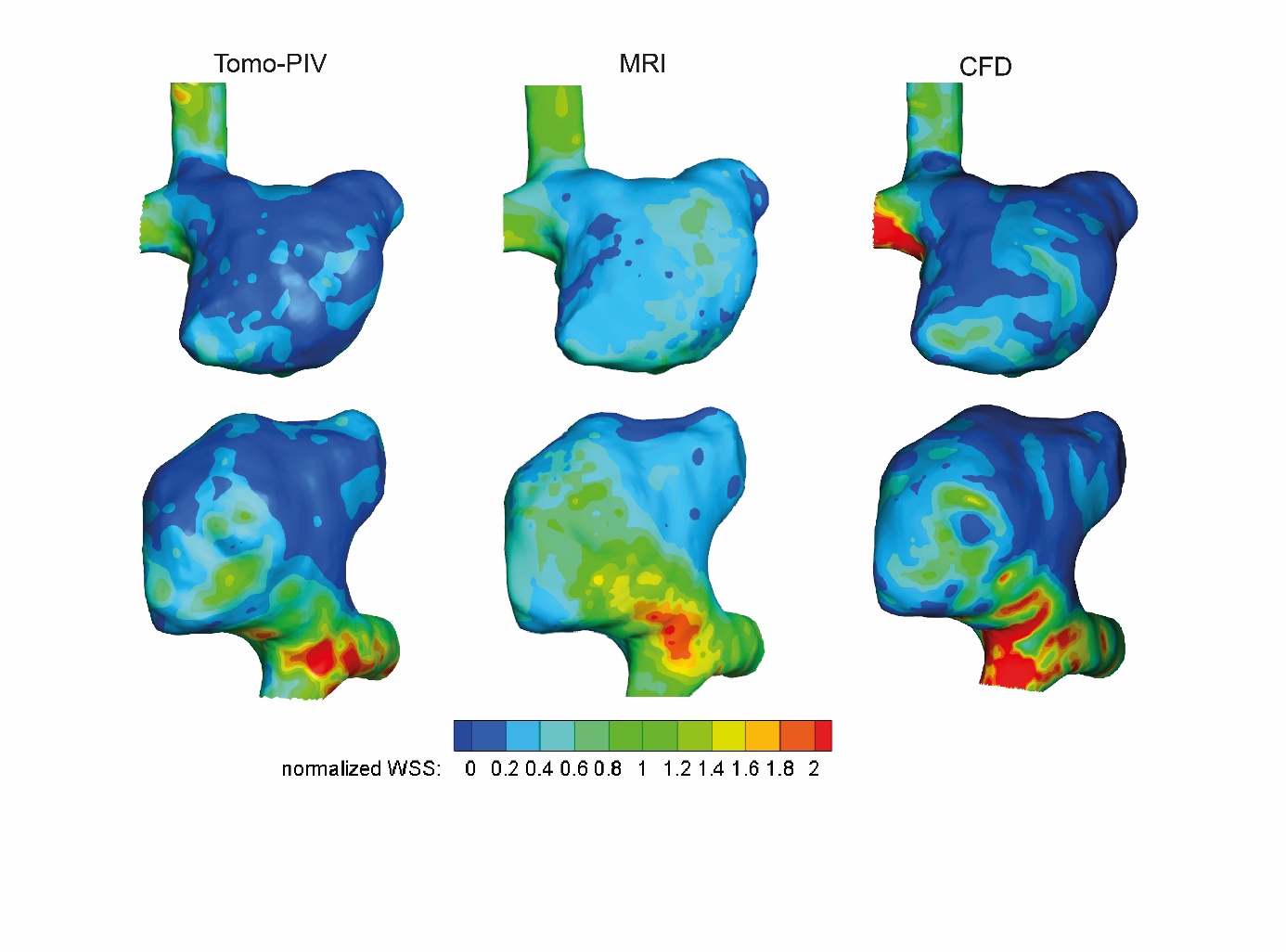


**Fig.4.** Normalized WSS comparison between Tomo-PIV, MRI and CFD at peak systole under (a) steady-state condition and (b) pulsatile flow condition.


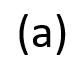

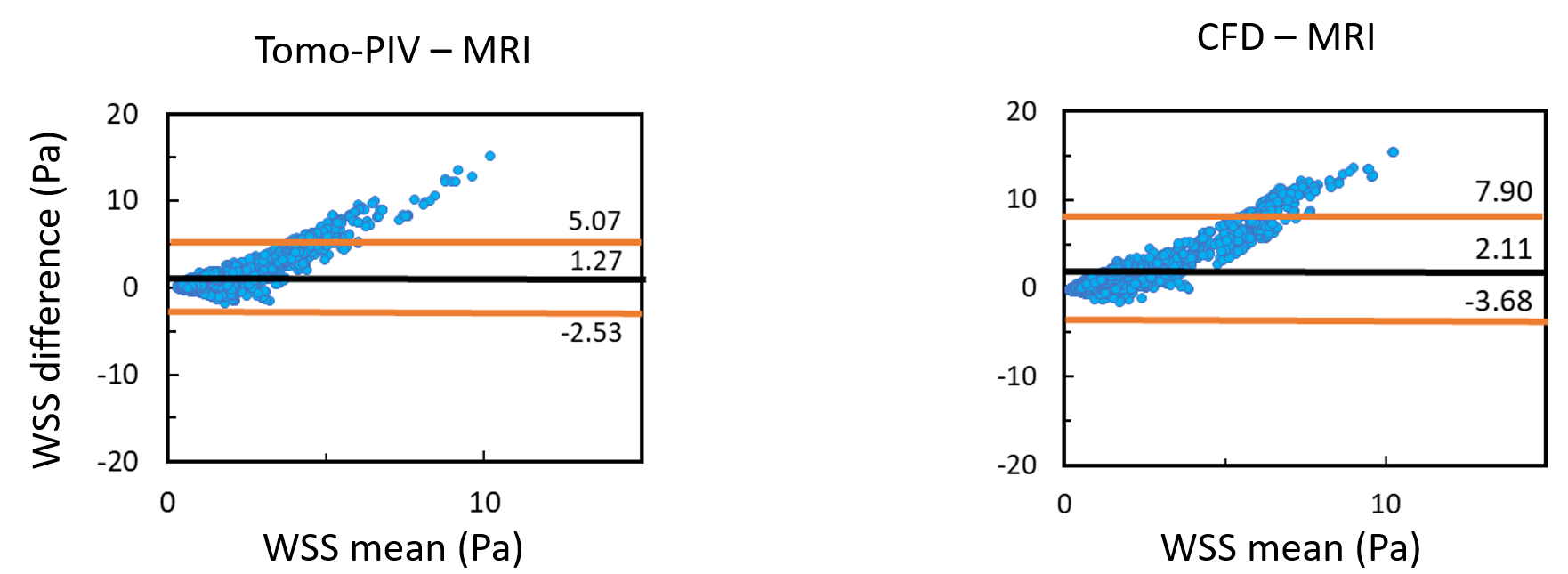


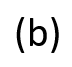

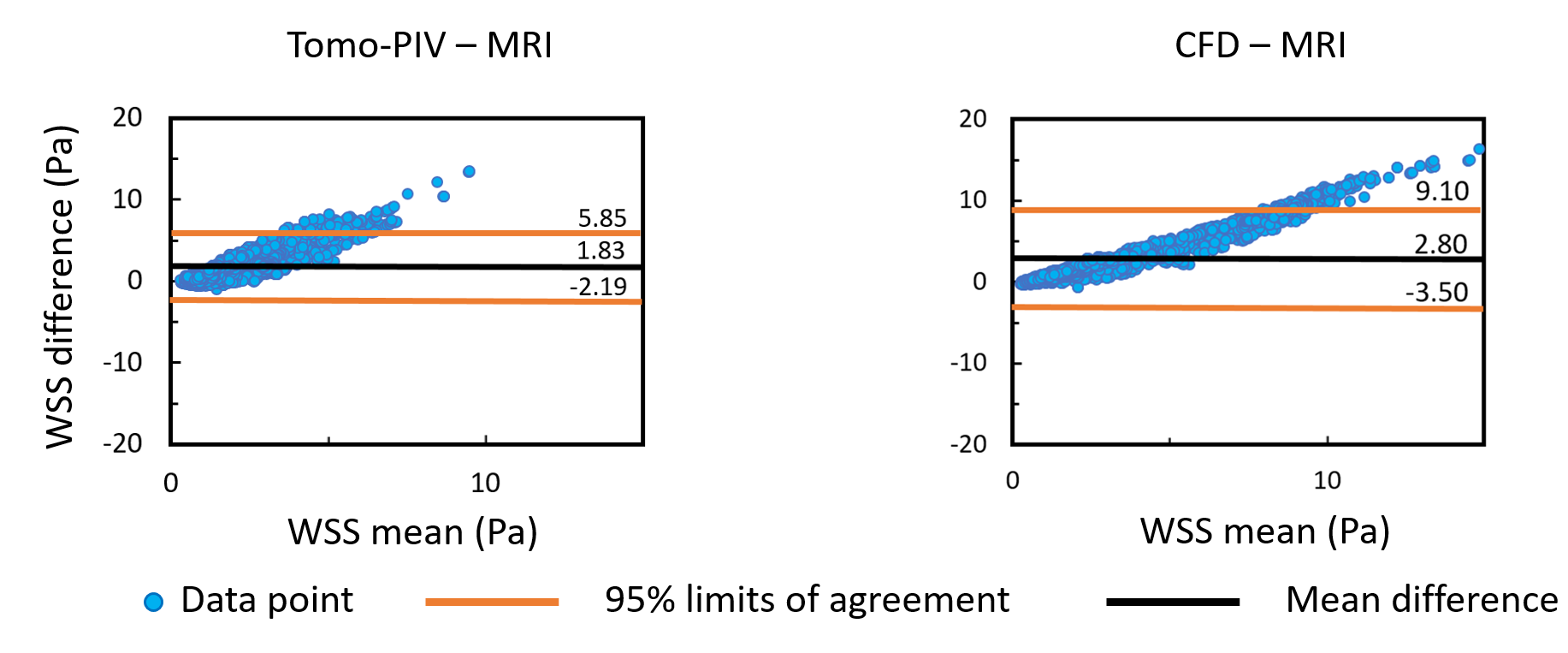


**Fig.5.** Bland-Altman plots of absolute WSS values at peak systole of (a) steady-state condition and (b) pulsatile flow condition, comparing MRI to Tomo-PIV and CFD
